# Redo Modified Bentall Procedure for Recurrent MRSA Complex Prosthetic Aortic Root Endocarditis Managed Successfully with Innovative Rapid Auto-Transfusion Technique: A Case Report

**DOI:** 10.12669/pjms.42.(11AASC).15803

**Published:** 2026-04

**Authors:** Abdul Ahad Sohail, Syed Shamair Ali Rizvi, Syed Shahabuddin, Abid Hussain, Shahid Ahmed Sami

**Affiliations:** 1 Dr. Abdul Ahad Sohail, FCPS. Section of Cardiothoracic Surgery, Department of Surgery, Aga Khan University Hospital, Karachi, Pakistan; 2 Syed Shamair Ali Rizvi Medical Student, Aga Khan University Hospital, Karachi, Pakistan; 3 Dr. Syed Shahabuddin, FCPS. Section of Cardiothoracic Surgery, Department of Surgery, Aga Khan University Hospital, Karachi, Pakistan; 4 Abid Hussain Chief Clinical Perfusionist, Department of Anesthesiology, Aga Khan University Hospital, Karachi, Pakistan; 5 Dr. Shahid Ahmed Sami, FRCS. Section of Cardiothoracic Surgery, Department of Surgery, Aga Khan University Hospital, Karachi, Pakistan

**Keywords:** Prosthetic valve endocarditis, MRSA, Redo Modified Bentall procedure, Aortic root replacement, Mechanical conduit, Multidrug resistance

## Abstract

**Background:**

Prosthetic valve endocarditis (PVE) remains one of the most challenging complications in cardiac surgery, particularly when caused by methicillin-resistant Staphylococcus aureus (MRSA). It carries high mortality and often mandates complex reoperative procedures. A multidisciplinary approach integrating surgical and antimicrobial strategies is crucial for favorable outcomes.

**Case Presentation:**

We report a 52-year-old man with a history of Stanford type A aortic dissection treated in 2013 with aortic root replacement using a 25 mm aortic valve and graft conduit and single-vessel coronary artery bypass grafting (CABG). Twelve years later, he presented with persistent fever and was diagnosed with MRSA prosthetic valve endocarditis unsuccessfully managed medically. Investigations revealed MRSA bacteremia, vegetations on the prosthetic conduit, and periaortic graft thickening. He underwent a high-risk redo Modified Bentall procedure with complete graft and valve explantation, radical debridement, and implantation of a 23 mm mechanical composite valve and graft conduit with saphenous vein reconstruction of the right coronary artery. Postoperatively, he developed complete heart block requiring permanent pacemaker implantation but recovered well on targeted antibiotics.

**Conclusion:**

This case illustrates the complexity of managing recurrent MRSA prosthetic valve endocarditis involving an aortic root graft and highlights that early multidisciplinary evaluation, aggressive surgical debridement, and appropriate antibiotic therapy remain the cornerstone of successful management. The use of a mechanical conduit provided a durable solution in a resource-limited setting without evidence of reinfection.

## INTRODUCTION

Prosthetic valve endocarditis (PVE) is a microbial infection of the heart that remains one of the most severe post operative complications after valve and root replacement surgery. Although it makes up just 20% of all infective endocarditis, it remains as one of the risky complications as seen by a high risk of perioperative mortality and recurrences in recent years.^1^,^2^ PVE has been seen to occur in around 6% of all patients with valve prostheses, with a notable increase in risk when the infection involves the aortic root or ascending grafts with perivalvular abscess formation, pseudoaneurysm, or fistula complicating the course and dramatically increasing both surgical complexity, morbidity and mortality.^3^

The decision to perform a redo aortic root replacement, in the setting of PVE is guided by the extent of infection (prosthetic dehiscence, root/graft involvement, abscess/fistula formation), persistent bacteremia despite antibiotics, presence of conduction abnormalities (suggesting root involvement), and hemodynamic instability or risk of embolic/septic complications.^4^

Herein, we report the case of a patient with prior aortic root replacement and coronary bypass grafting who developed recurrent MRSA PVE involving the aortic root and graft complex, requiring high-risk redo aortic root replacement.

## CASE PRESENTATION

A 52-year-old man with a past medical history of Stanford type A acute aortic dissection complicated by severe aortic regurgitation and right coronary artery ostia dissection had undergone emergency aortic root replacement with a 25 mm mechanical aortic valve conduit and a single-vessel coronary artery bypass graft with Reverse Saphenous Vein Graft (RSVG) to Right Coronary Artery (RCA) in 2013 and remained on lifelong anticoagulation with warfarin and regular follow-up in the cardiology clinic.

Twelve years later, he presented with a three-week history of intermittent high-grade fever, chills, malaise, and reduced appetite. Initial laboratory investigations revealed normocytic anemia with a hemoglobin of 9.6 g/dL, leukocytosis of 15.4 × 10^9^/L, and elevated inflammatory markers. Two sets of blood cultures grew methicillin-resistant *Staphylococcus aureus* (MRSA). A transesophageal echocardiogram (TEE) demonstrated a well-seated prosthetic valve without definite vegetations or perivalvular leak. A diagnosis of early prosthetic valve endocarditis (PVE) was made and the patient was started on intravenous antibiotics for eight weeks.

However, after a brief afebrile interval, he developed recurrent high-grade fever associated with chills, rigors, and progressive dyspnea on exertion for 2 weeks. On readmission to our institute, he appeared acutely ill but alert and oriented and hemodynamically stable. Examination revealed multiple Janeway lesions on his palms and soles.

Laboratory testing now showed worsening anemia (hemoglobin 7.9 g/dL), leukocytosis (TLC 21.6 × 10^9^/L) and markedly elevated CRP (211 mg/L). Serum creatinine had risen to 1.9 mg/dL, his INR was supratherapeutic at 7.9, later corrected to 2.2 with vitamin K and withholding of warfarin. Blood cultures again grew MRSA on multiple occasions.

**Fig.1:**
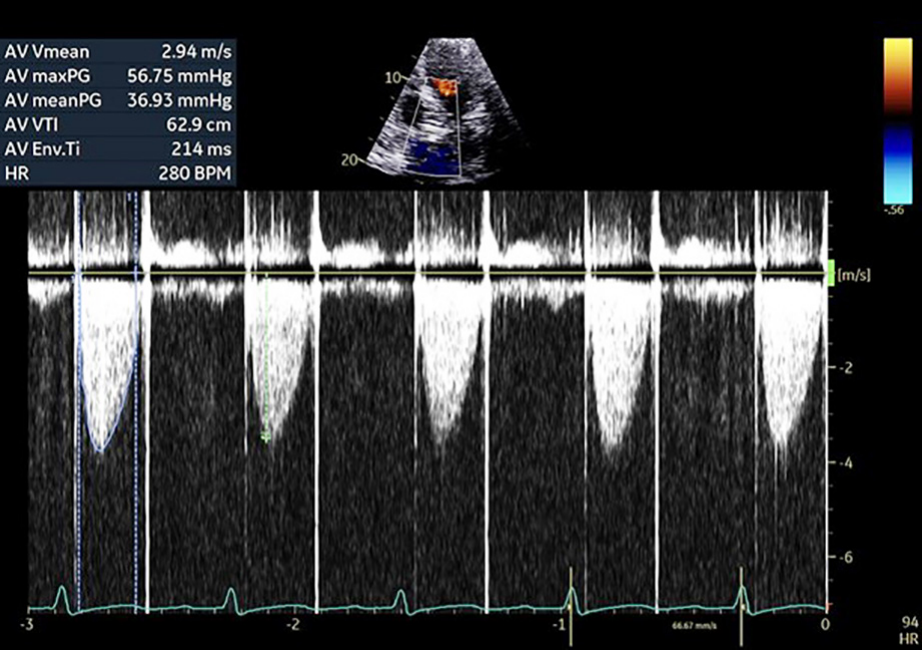
Echocardiography demonstrating increased aortic valve gradients and velocity.

**Fig.2:**
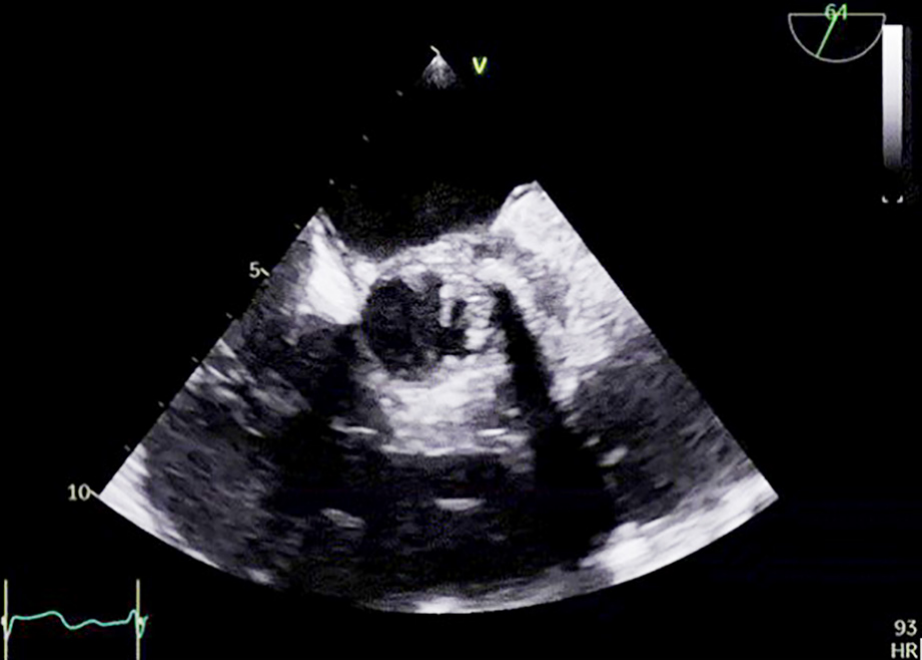
Transesophageal echocardiogram, showing aortic graft vegetations and peri-aortic graft thickening.

**Fig.3:**
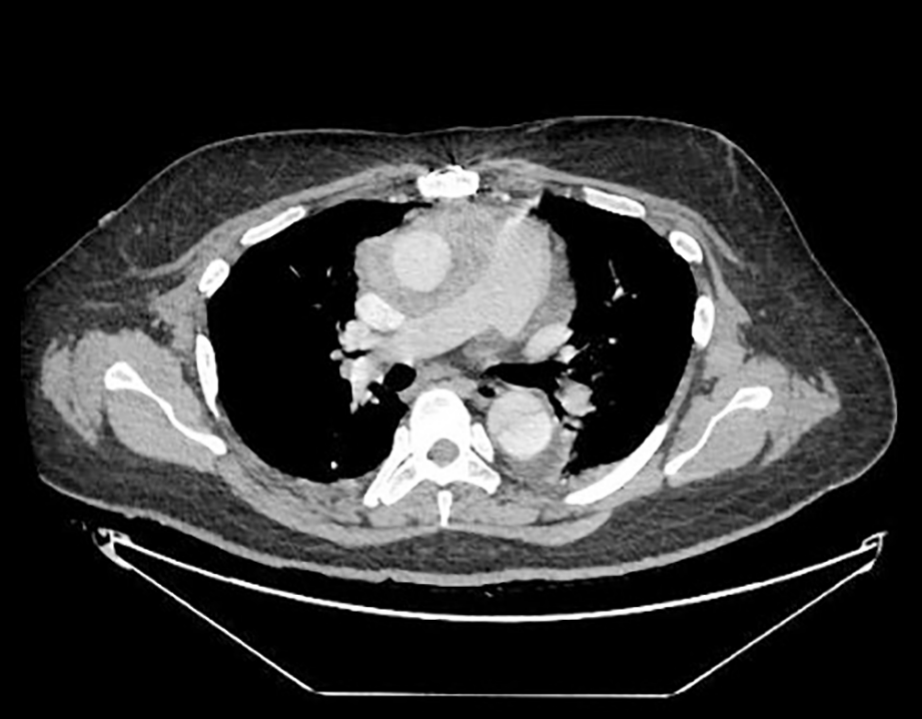
Computed tomography scan showing inflammation and thickening surrounding the ascending aortic graft.

Repeat TEE (Fig. 1 and 2) revealed multiple mobile vegetations attached to the prosthetic valve leaflets and the conduit wall, with mild paravalvular leak and evidence of periaortic root thickening suggestive of an abscess. Contrast-enhanced CT (Fig.3) of the thorax demonstrated soft-tissue thickening and inflammatory changes around the ascending aortic graft consistent with infection extending into the periaortic region. The multidisciplinary team—comprising cardiology, infectious disease, and cardiothoracic surgery—recommended urgent reoperative aortic root replacement.

After preoperative optimization and continuation of broad-spectrum intravenous antibiotics (meropenem, vancomycin, and gentamicin), the patient underwent a high-risk redo Modified Bentall procedure. Femoral artery and vein were exposed and purse string sutures were taken. Redo-Sternotomy was then done. Dense adhesions were encountered between the pericardium and epicardium. During dissection of ascending aorta, sudden massive bleeding was encountered as previous RSVG to RCA graft was dislodged from the ascending aorta leading to hemodynamic instability. Systemic heparinization was done immediately with 400 international units per kilogram. To rapidly restore circulating volume and maintain hemodynamic stability, an innovative rapid auto-transfusion technique was employed. A sterile 150-cm length, 4.0-mm diameter line was used to connect the purge line of the extracorporeal circuit to the central venous pressure (CVP) line. This configuration enabled direct reinfusion of shed blood through the roller pump, allowing prompt hemodynamic recovery with minimal resources and time. Meanwhile cardiopulmonary bypass was established via right femoral arterial (23 Fr) and venous (25 Fr) cannulation. Systemic cooling was performed to a target temperature of 28°C, and four units of packed red blood cells were administered to maintain the desired hematocrit level. Vacuum-assisted venous drainage (−20 to −40 mmHg) was utilized to optimize venous return, while conventional ultrafiltration aided in maintaining intravascular volume and hematocrit balance. Mean arterial pressure was maintained between 60–70 mmHg with continuous metabolic and temperature monitoring.

**Fig.4:**
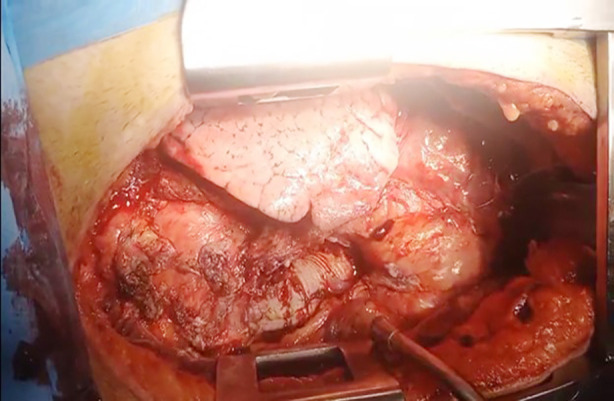
Post-operative image of prosthetic graft conduit (blue arrow) and proximal anastomosis of RSVG to RCA on dacron graft conduit (yellow arrow).

Aortic cross clamp was applied and aortotomy was done proximally. Antegrade cardioplegia was given directly into the coronary ostia and diastolic arrest was achieved. Purulent discharge was noted in the periaortic space and sent for microbiological analysis. The prosthetic valve and conduit were found to be heavily colonized with vegetations and surrounded by fibrotic tissue and pockets of abscess. Complete explantation of the infected prosthesis and extensive debridement of necrotic tissue were performed. A 23mm Medtronic mechanical composite graft (Fig.4) was then anastomosed to the left ventricular outflow tract using interrupted pledgeted 2-0 Ethibond sutures with overlapping technique. The left coronary button was directly reimplanted, and the prior saphenous vein graft to right coronary artery was reconstructed using a new saphenous vein segment (end-to-side anastomosis to the previous saphenous vein graft and proximal re-anastomosis to the new aortic conduit).

He was weaned from cardiopulmonary bypass with pacing support due to complete heart block and transferred to the cardiac intensive care unit with an open chest and mediastinal packing. Serial mediastinal washouts were performed, and the chest was definitively closed 48 hours later. He was extubated the following day. Persistent complete heart block necessitated dual-chamber permanent pacemaker implantation on postoperative day-7 after clearance from the infectious disease team. The patient developed transient acute kidney injury, which resolved with conservative management. Postoperative blood cultures were sterile, while tissue and pus cultures from the graft showed heavy growth of MRSA sensitive to vancomycin and gentamicin. Fungal and mycobacterial tuberculous studies were negative.

A postoperative transthoracic echocardiogram demonstrated normal left ventricular size and systolic function with an ejection fraction of 55%, a well-seated mechanical prosthesis at the aortic position, a peak transvalvular gradient of 28 mmHg, mean gradient of 15 mmHg and no regurgitation. The patient completed a six-week course of intravenous vancomycin and gentamicin via a peripherally inserted central catheter (PICC) line, maintaining therapeutic INR levels on warfarin. He was discharged in stable condition after 20 days of hospitalization.

At his three-month follow-up, he remained afebrile and asymptomatic, with stable pacemaker function, normalized inflammatory markers (CRP 12 mg/L), and echocardiographic evidence of a well-functioning prosthesis without paravalvular leak or recurrent infection.

## DISCUSSION

Staphylococcus Aureus causing prosthetic valve endocarditis (PVE) remains one of the most serious complications following cardiac valve surgery, with it accounting for around 16.7% of all PVE cases seen globally.^5^ Mortality remains high, particularly when infection involves Methicillin Resistant *Staphylococcus aureus* species or has prosthetic aortic root involvement, where destructive infection can rapidly progress to annular abscess, pseudoaneurysm, or fistula formation.^6^ Among these, *methicillin-resistant Staphylococcus aureus* (MRSA) PVE is associated with some of the poorest outcomes, with reported in-hospital mortality rates around 47.5% despite optimal medical therapy.^7^

Transesophageal echocardiography (TEE) remains the diagnostic gold standard, while computed tomography and fluorodeoxyglucose positron emission tomography (FDG-PET/CT) are increasingly employed to detect perivalvular and graft involvement in equivocal cases.^8^ Also attempts should be made to elucidate presence of underlying immune deficiency which was not done in our case.

The “cornerstone of therapy” has said to employ the prolonged antibiotic course combined with surgical debridement of the infected prostheses because medical therapy alone is rarely sufficient when structural complications such as abscess, fistula, or prosthetic dehiscence are involved. Indications for early surgery include heart failure, uncontrolled infection despite appropriate antibiotics, embolic phenomena, and infection involving prosthetic material or grafts.^9^

Redo aortic root replacement (Modified Bentall procedure) in the context of active infection remains one of the most technically demanding operations in cardiothoracic surgery. In the presence of periaortic abscess or conduit infection, complete excision of the infected prosthesis and radical debridement of all devitalized tissue are mandatory.^9^

This case also underscores the critical importance of a multidisciplinary approach in managing complex prosthetic valve endocarditis, particularly in an era of rising antimicrobial resistance and changing pathogenic patterns in infective endocarditis.^10^

## CONCLUSION

Recurrent MRSA prosthetic valve endocarditis involving the aortic root represents a rare but catastrophic condition demanding prompt recognition and multidisciplinary management. While medical therapy is the initial step, definitive cure in cases of prosthetic or graft involvement relies on radical surgical debridement and replacement. Redo aortic root replacement, though technically challenging, can be performed safely with meticulous preoperative planning, aggressive infection control, and close postoperative monitoring. Our experience reinforces that timely surgery remains the cornerstone of management for complex aortic root infections and can lead to successful outcomes even in high-risk reoperative settings.

### Author Contributions:

**AAS:** Conceptual and design of the manuscript, acquisition and interpretation of the data, drafting of the manuscript and critical revision.

**SSAR:** Conceptual and design of the manuscript, acquisition and interpretation of the data, drafting of the manuscript.

**SS:** Conceptual and design of the manuscript, acquisition and interpretation of the data, drafting of the manuscript and critical revision.

**AH:** Conceptual and design of the manuscript, acquisition and interpretation of the data, drafting of the manuscript. (Also since cardiopulmonary bypass machine was used so all the data regarding it was acquired and interpreted by him as he is the chief perfusionist.

**SAS:** Conceptual and design of the manuscript, acquisition and interpretation of the data, drafting of the manuscript and critical revision. Final approval.

All authors have approved the final version of the manuscript and are accountable for the integrity of the study.
